# Effects of transitional care on self-care, readmission rates, and quality of life in adult patients with systemic lupus erythematosus: a randomized controlled trial

**DOI:** 10.1186/s13075-018-1670-4

**Published:** 2018-08-16

**Authors:** Xia Xie, Yuqing Song, Hui Yang, Anliu Nie, Hong Chen, Ji-ping Li

**Affiliations:** West China School of Nursing & Department of Nursing, West China Hospital, Sichuan University, No.37 Guoxue Xiang, Chengdu, Sichuan 610041 China

**Keywords:** Systemic lupus erythematosus, Transitional care, Self-care, Readmission, Quality of life

## Abstract

**Background:**

Lack of adequate self-care, frequent admissions, and poor quality of life are common and serious problems in adult patients with systemic lupus erythematosus (SLE). Some studies have revealed that transitional care is effective in improving self-care and quality of life as well as reducing rehospitalization rates. However, limited studies explored its effects in adult patients with SLE. Therefore, we performed a study to examine the effects of transitional care on self-care, readmission rates, and quality of life in adult patients with SLE.

**Methods:**

This study was a single-center, single-blind, and parallel-group randomized controlled trial comparing transitional care with usual care in SLE patients from a university hospital in China. Evaluations were conducted at baseline before discharge and at 3 months after discharge by using hospital readmission rate, the Exercise of Self-Care Agency Scale, and the Medical Outcomes Study Short Form 36-item Health Survey for self-care and quality of life. Data were collected between June and December 2016.

**Results:**

Compared with the usual care group, the transitional care group reported significantly greater improvement in self-care and quality of life. Additionally, the 30-day readmission rate for the patients in the transitional care group was significantly lower than in the usual care group, and this effect remained significant at 60 and 90 days after patient discharge.

**Conclusion:**

This study shows that transitional care improves self-care and quality of life in adult patients with SLE and reduces readmissions. However, further studies are needed.

**Trial registration:**

China clinical trial registry, ChiCTR-IPR-16007708. Registered January 5, 2016.

**Electronic supplementary material:**

The online version of this article (10.1186/s13075-018-1670-4) contains supplementary material, which is available to authorized users.

## Background

Systemic lupus erythematosus (SLE) is a common chronic autoimmune inflammatory disease. It is thought that about 50,000 people in the UK [1] and between 161,000 and 322,000 in the USA [2] are afflicted with SLE. Nor is SLE a rare disease in China, where the estimated prevalence is 30 to 100 cases per 100,000 in adults [3, 4]. In recent decades, the survival rate of patients with SLE has improved because of improvements in SLE diagnosis and treatment [5]. However, nearly 60% of patients with SLE had episodes of flare or persistently active disease per year [6]. This is especially unfortunate because patients with active SLE usually experience more serious physical and psychosocial problems [7], resulting in frequent admissions [8–10]. Previous studies have shown that approximately 16.5% of patients hospitalized for SLE are readmitted within 30 days because of the development of new or worsening symptoms [8], and about 35% within 1 year after discharge [9, 10]. In total, compared with those with other chronic illnesses, patients with SLE have the sixth highest hospital readmission rate in the USA [11]. The frequent admission significantly aggravates the burden of patients with SLE. Anandarajah et al. [12] found that the total cost of SLE was $3,971,799 in 2015 but that the total cost for all readmissions among those with a confirmed SLE diagnosis was $1,687,450 and the cost for those readmitted within a month was calculated to be $1,036,438. In addition to the expense, frequent admissions have considerable adverse effects on patients’ quality of life [13]. Khanna et al. [14] found that quality of life of patients with SLE was poor and their quality-of-life scores were lowest in the exacerbation period.

Previous studies have shown that adequate self-care knowledge and skill are key factors for patients to reduce readmission rates and improve quality of life [15, 16]. However, lack of self-care knowledge and skills are common in patients with SLE [17–19]. Sullivan [20] developed a questionnaire to investigate disease and self-care knowledge of patients with SLE, and only 13.4% of the participants scored 50% or higher on the questionnaire. Therefore, it is urgent to develop targeted intervention to improve self-care, reduce readmission rates, and enhance quality of life in patients with SLE.

Transitional care is a set of actions designed to ensure the coordination and continuity of health care when patients transfer between different settings (e.g., from hospital to home). Previous studies have shown that it is an effective model to improve self-care, reduce rehospitalization rates, and enhance quality of life of patients with other chronic conditions, mainly heart failure and diabetes [21–23]. Some transitional care studies in SLE have also been published in recent years, but most focused on the transition from child-centered to adult-oriented care [24]. Studies evaluating transition care in adult SLE are scarce, and the reproducibility and effectiveness of transitional care in adult patients with SLE remain unclear. However, the incidence of SLE peaked in child-bearing age [25]. So we chose adult patients with SLE as subjects of the study. We hypothesized that transitional care would improve self-care, decrease readmission rate, and enhance quality of life in this complex patient population.

## Methods

### Study design

This study was a single-center, single-blind, and parallel-group randomized controlled study. It was approved by the West China Hospital Medical Ethics Committee (ID 20160041), and written informed consent was obtained from all participants.

### Participants

The sample size of this study was calculated on the PASS 11 (NCSS Statistical Software, Kaysville, UT, USA) on the basis of the self-care baseline and 12th-week data (94.7 ± 9.2 and 111.6 ± 9.8, respectively), as revealed in the pilot study. To achieve a power of 80% with a two-tailed alpha of 0.05, 56 subjects per group were needed. Given a dropout rate of 20%, 136 eligible participants were required.

Patients were recruited during their hospital admission to the Department of Rheumatology of the West China Hospital, Sichuan University. To be included in the study, participants had to be at least 18 years old, be diagnosed with SLE, be admitted because of the development of new or worsening SLE-related symptoms, be discharged from hospital to home, have sufficient cognitive ability to communicate (determined by researcher), have access to a telephone, and be willing to participate in the study. Patients who were participating in another intervention study were excluded from the study.

### Randomization, allocation, and blinding

Before the research began, a researcher (HC) generated a randomization sequence to assign participants to the transitional care group or the usual care group. The sequential numbered, opaque sealed envelopes were used to conceal the allocation sequence until participants provided written informed consent and baseline information; another researcher (XX) opened the envelope in each patient’s presence and revealed the result of allocation. In other words, all researchers and participants in this study were not blinded to the allocation, but the investigators were.

### Intervention

#### Transitional care

Chow and Wong [26] have suggested that a successful transitional care program for patients with chronic conditions should be based on a comprehensive assessment of patient needs. The Omaha System, a framework for problem-solving, can provide a good way to describe the primary needs of patients and interventions that solve patient problems [27]. The system consists of three subsystems: problem classification scheme, intervention scheme, and problem rating scale. The problem classification scheme covers physiological, psychosocial, health-related behaviors and four environmental domains, each of which contains a number of health problems, totaling 42 health problems. The intervention scheme consists of teaching, guidance, and counseling, treatments and procedures, case management and four broad surveillance categories. The problem rating scale is composed of three Likert 5-point scales to measure client’s knowledge, behavior, and status [28]. In this study, the transitional care was designed on the basis of the Omaha System.

First, based on the problem classification scheme as well as a literature review and experts’ advice, 23 SLE-related health problems were included in the transitional care protocol in the domains of physiological (9), psychosocial (4), health-related behaviors (8), and environmental (2). Each of the problems can be scored on knowledge, behavior, and status by using the three Likert 5-point scales in the problem rating scale. If any one of the scores of a patient’s knowledge, behavior, or status is less than 4 points, it indicates that the intervention categories and specific interventions corresponding to the problem should be provided [29, 30]. The intervention categories and specific interventions corresponding to each problem in the study were established by the research teams on the basis of the Omaha System intervention scheme and a review of literature and were revised by two experienced rheumatologists and three clinical nurse specialists. The Omaha System assessment-intervention framework is presented in Additional file 1: Table S1.

The duration of the transitional care was 12 weeks. It consisted of four structural assessments and corresponding interventions as well as four telephone follow-ups (Table 1). All interventions were delivered by two nurses with a master’s degree who were experienced in the nursing of SLE. Both nurses were well trained in transitional care and had Omaha System knowledge and skills. Additionally, the nurses were supported by one doctor and three nursing specialists in the Department of Rheumatology, as appropriate.

**Table 1:** Transitional care plan

Category	Time	Implementation method
Structural assessments and corresponding interventions designed on the basis of the Omaha System	1, 4, 8, and 12 weeks after discharge	Face-to-face contact with the participants. A client-centered approach was used to address patients’ existing or potential health problems. First, identify patients’ existing or potential health problems: 23 systemic lupus erythematosus–related health problems were scored on knowledge, behavior, and status of disease management by using the three Likert 5-point scales. Second, provide corresponding interventions: If any one of the scores of a patient’s knowledge, behavior, or status is less than 4 points, the intervention categories and specific interventions corresponding to the problem would be provided.
Telephone follow-up	2, 3, 6, and 10 weeks after discharge	Phone counseling was conducted to identify and discuss any barriers and concerns which the patient may have regarding their disease.

### Usual care

For the usual care group, no structured educational or supportive post-discharge care was provided. Instead, patients received brief instructions on medications and basic health advice when they collected prescribed medications on hospital discharge.

### Outcome measures

All participants completed three questionnaires. The baseline demographic survey included age, gender, education level, marital status, employment status, medical insurance, diagnosis duration of SLE, hospitalizations (past 1 year), and comorbidity. The other two instruments comprised measures of self-care and quality of life. Two well-trained investigators, who had no information about subjects’ group allocations, conducted face-to-face interviews to collect the abovementioned indicators just before hospital discharge and 12 weeks after discharge. Additionally, the two investigators completed all participants’ baseline disease activity questionnaires and collected data on participants’ readmissions within 30, 60, and 90 days on the basis of the information provided by hospital records and the patients.

#### Disease activity

The Systemic Lupus Erythematosus Disease Activity Index 2000 (SLEDAI-2K) was used to measure disease activity of participants. The SLEDAI-2K includes 24 weighted objective clinical and laboratory variables. The sum of the scores of all 24 variables is the total score of the SLEDAI-2K. The total score of the SLEDAI-2K ranges from 0 to 150, and a higher score indicates higher disease activity [31].

#### Self-care

Self-care of patients was assessed by the Exercise of Self-Care Agency Scale (ESCA). The scale was developed by Kearney and Fleischer [32], and Wang and Laffrey [33] translated it into Chinese. The scale has 43 items, which consist of four dimensions: self-concept, motivation, knowledge and information seeking, and passivity. Each item is rated on a 5-point Likert scale. The total score of the ESCA ranges from 0 to 172, and a higher score represents higher self-care capability. Previous Chinese validity and reliability studies for the ESCA have reported that Cronbach’s α coefficient and content validity ranged from 0.86 to 0.91 and from 0.8 to 0.92, respectively [33, 34]. The internal consistency coefficient of the scale in this study was 0.85.

#### Quality of life

The Medical Outcomes Study Short Form 36-item Health Survey (SF-36) was used to assess quality of life. The SF-36 contains eight domains, which can be further summarized into two component scores: physical component summary (PCS) and mental component summary (MCS). After recoding, the two components both can reach a maximum of 100, and a higher score represents better quality of life. The SF-36 has been tested and recommended for use in patients with SLE [35]. The Cronbach’s α coefficient of the scale in this study was 0.88.

### Statistical analysis

Descriptive statistics (means, standard deviations, frequencies, and percentages) were used to summarize the characteristics of participants. The baseline characteristics of the two groups were compared by using the chi-squared test or the Student *t* test, as appropriate. The chi-squared test was also used to examine the effect of intervention on readmission rates. Additionally, the effects of interventions on self-care and quality of life were examined by the analysis of covariance (ANCOVA). All data analyses were performed by using SPSS 21.0 (SPSS Inc., Chicago, IL, USA), and a *P* value of less than 0.05 was considered statistically significant.

## Results

### Demographic and clinical characteristics

**Fig. 1:**
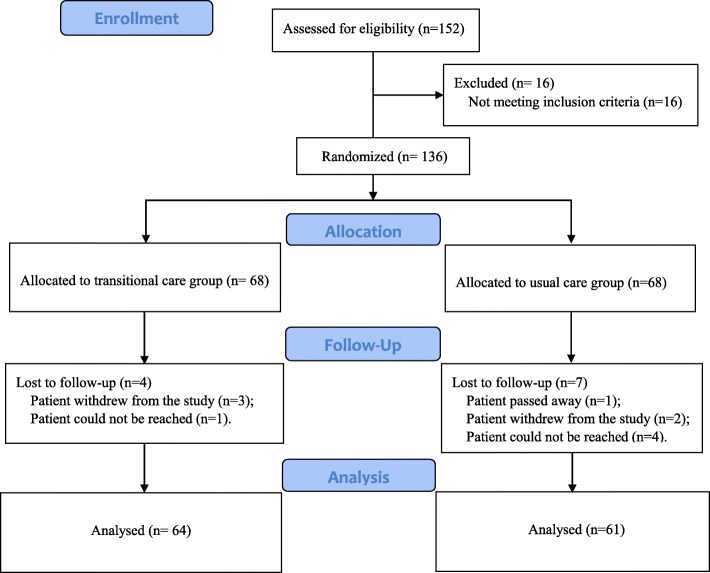
Consort diagram of participant flow

In the study, 136 eligible patients were recruited, but four cases in the intervention group and seven in the control group were lost to follow-up. Finally, a total of 125 participants (64 in the intervention group and 61 in the control group) completed all waves of the study (Fig. 1).

Table 1 presents the baseline characteristics of patients. The average age of patients was 37.1 (14.1) years, and 88.8% were female. The majority of patients were married. Nearly half of the cases had a high school education or less, and most were unemployed. Two thirds of patients have medical insurance. More than half of the patients had SLE that was diagnosed less than three years ago, had an average SLEDAI-2K score of 10.3, and were hospitalized in the past year. Also, the great majority of patients had comorbidity, and nephritis and hypertension were the most common. In terms of the characteristics of therapeutic regimen, more than half of the patients received glucocorticoid combined with hydroxychloroquine.

There were no differences in demographic or clinical characteristic variables, self-care, or quality of life between the intervention group and comparison group at baseline according to the *t* test and chi-squared analysis (Table 2).

**Table 2:** Participants’ characteristics and differences among these variables between the two groups at baseline (*N* = 125)

Variable	Total	Control group (*n* = 61)	Intervention group (*n* = 64)	*t*	*P* value
	Mean (SD)	Mean (SD)	Mean (SD)		
Age, years	37.1 (14.1)	38.4 (15.8)	35.9 (12.3)	1.015	0.312
SLEDAI-2K	10.3 (4.4)	9.7 (3.8)	10.9 (4.9)	−1.584	0.116
Variable	*n* (%)	*n* (%)	*n* (%)	*χ* ^*2*^	*P* value
Gender					
Male	14 (11.2)	7 (11.5)	7 (10.9)	0.009	0.924
Female	111 (88.8)	54 (88.5)	57 (89.1)		
Education level					
High school or less	60 (48.0)	30 (49.2)	30 (46.9)	0.066	0.797
Above high school	65 (52.0)	31 (50.8)	34 (53.1)		
Marital status					
Married	92 (73.6)	47 (77.0)	45 (70.3)	0.729	0.393
Single	33 (26.4)	14 (23.0)	19 (29.7)		
Work status					
Employed	27 (21.6)	12 (19.7)	15 (23.4)	0.261	0.609
Unemployed	98 (78.4)	49 (80.3)	49 (76.6)		
Medical insurance					
Yes	82 (65.6)	39 (63.9)	43 (67.2)	0.146	0.702
No	43 (34.4)	22 (36.1)	21 (32.8)		
Diagnosis duration					
≤3 years	63 (50.4)	30 (49.2)	33 (51.6)	0.071	0.790
>3 years	62 (49.6)	31 (50.8)	31 (48.4)		
Hospitalizations (past 1 year)					
Yes	81 (64.8)	40 (65.6)	41 (64.1)	0.031	0.860
No	44 (35.2)	21 (34.4)	23 (35.9)		
Comorbidity					
Yes	109 (87.2)	51 (83.6)	58 (90.6)	1.378	0.240
No	16 (12.8)	10 (16.4)	6 (9.4)		
Type of SLE medicine					
GC	21 (16.8)	11 (18.0)	10 (15.6)	0.844	0.839
GC plus HCQ	65 (52.0)	32 (52.5)	33 (51.6)		
GC plus ISD	20 (16.0)	8 (13.1)	12 (18.8)		
GC plus HCQ plus ISD	19 (15.2)	10 (16.4)	9 (14.1)		

Abbreviations: *GC* glucocorticoids, *HCQ* hydroxychloroquine, *ISD* immunosuppressive drug, *SD* standard deviation, *SLE* systemic lupus erythematosus, *SLEDAI-2K* Systemic Lupus Erythematosus Disease Activity Index 2000

### Effects of transitional care on outcomes

The ANCOVA results show that the intervention group reported significantly greater improvement in total self-care score and score of its four subscales (self-concept, motivation, knowledge and information seeking, and passivity). Also, compared with the control group, the intervention group had significantly greater improvement in SF-36 MCS and SF-36 PCS over the course of 3 months (Table 3). Additionally, the 30-day readmission rate for patients in the intervention group was significantly lower than in the control group, and this effect remained significant at 60 and 90 days after patient discharge (Table 4).

**Table 3:** Effects of transition care on self-care and quality of life among SLE patients (*N* = 125)

Variables	Control group (*n* = 61)		Intervention group (*n* = 64)		*F*	*P* value
	Pre-test Mean (SD)	Post-test Mean (SD)	Pre-test Mean (SD)	Post-test Mean (SD)		
Self-care agency						
Total score	95.7 (9.6)	100.9 (8.5)	92.9 (10.8)	112.9 (6.8)	162.978	< 0.001
Self-concept	15.8 (3.5)	15.7 (2.3)	15.4 (3.4)	18.9 (1.9)	173.321	< 0.001
Motivation	17.3 (4.0)	18.9 (1.2)	16.5 (1.3)	21.6 (0.7)	251.959	< 0.001
Knowledge	33.9 (4.5)	37.9 (3.9)	33.2 (5.5)	41.4 (3.6)	55.925	< 0.001
Passivity	28.7 (2.5)	28.5 (2.7)	27.8 (2.9)	31.0 (1.9)	67.825	< 0.001
Quality of life						
SF-36 PCS	50.0 (11.9)	60.5 (12.3)	48.8 (12.4)	63.7 (10.9)	4.061	0.046
SF-36 MCS	49.8 (13.1)	57.4 (9.3)	45.4 (14.3)	61.1 (9.1)	11.438	0.001

Abbreviations: *SD* standard deviation, *SF-36 MCS* mental component summary of the Medical Outcomes Study Short Form 36-item Health Survey, *SF-36 PCS* physical component summary of the Medical Outcomes Study Short Form 36-item Health Survey

**Table 4:** Effect of transition care on readmission rates among patients with systemic lupus erythematosus (N = 125)

Variables	Control group (*n* = 61)	Intervention group (*n* = 64)	*χ* ^*2*^	*P* value
30-day readmissions	13 (21.3)	3 (4.7)	7.733	0.005
60-day readmissions	16 (26.2)	7 (10.9)	4.864	0.027
90-day readmissions	17 (27.9)	9 (14.1)	4.611	0.032

## Discussion

This study examined the effects of transitional care in adult patients with SLE. The transitional care was planned on the basis of the Omaha System as well as a literature review and experts’ advice. It consisted of four structural assessments and corresponding interventions as well as four telephone follow-ups. The results showed that the transitional care group experienced more improvement in self-care and quality of life. Moreover, the transitional care group had a lower rate of rehospitalization compared with the usual care group.

First, the results from this study support the hypothesis that transitional care was an effective model to improve self-care of patients with SLE. This result was consistent with previous studies in other chronic diseases. For example, the study by Hull [36] assessed the impact of a transitional care model in patients with heart failure and found a greater improvement in participants’ self-care in the transitional care group. A possible reason for the significant effect of transitional care on self-care is that the strategies in this model could motivate patient initiative, as recommended by previous research [37]. In our study, the Omaha System was adopted to identify patients' existing or potential health problems and to develop targeted interventions. Also, telephone follow-up was used to monitor participant compliance with transitional care interventions and identify the self-management barriers of patients. These interventions might be helpful in building positive patient-staff relationships, supporting patients’ individual care needs, motivating patient initiative and activity, and enhancing patient compliance with transitional care interventions, thereby improving patients’ self-care.

Second, there was a significant reduction in readmissions of patients with SLE during the transitional care program. This is similar to the study by Verhaegh et al. [38], who conducted a systematic review of 26 randomized controlled trials of transitional care among patients with heart failure. The authors found that transitional care was effective in reducing intermediate-term (31–180 days) readmissions and that only high-intensity models were effective in reducing short-term (30 days or less) readmissions. Simultaneously, they pointed out that the reduced readmission rate was significantly associated with care coordination by nurses during early post-discharge period. The result was supported by previous research which suggested that lack of timely follow-up arrangements and poor communication between health-care providers and patients in the first week after discharge contribute to frequent readmission [39]. In our study, patients received four structural assessments and corresponding interventions as well as four telephone follow-ups, and the first session was at the first week after patients’ discharge, ensuring a high-intensity continuity of health care during the transition period. In addition, Gray et al. [40] conducted a study of the theory of transitional care and suggested that transitional care could reduce patients’ hospitalizations by improving interpersonal communication and the disease management abilities and behaviors of patients. This is in line with our research hypothesis that transitional care may improve patients’ self-care and thus reduce their readmissions.

Last but not least, in terms of the effects of transitional care on quality of life among different disease populations, the results are controversial. Some studies suggested that transitional care was effective in improving quality of life of patients with chronic conditions [41], whereas others did not reveal a similar result [42]. The present study provides evidence for transitional care as an effective way to improve quality of life for adult patients with SLE. The effectiveness of transitional care in improving quality of life may be attributed to the characteristics of the strategies adopted, including patient-centered assessments and interventions and telephone follow-up counseling. The strategies in this study might provide informational and emotional support to help patients to understand and grasp the skills and knowledge on how to become involved in controlling SLE symptoms and improving physical and mental health.

### Implications for general practices and future research

This study is timely and contributes to the evidence that transitional care may improve self-care, reduce readmissions, and improve quality of life of adult patients with SLE. These findings might be highly relevant for policy makers and health-care providers because of the increasing trend of patients with SLE and the heavy burden of the disease [4, 7]. In view of the feasibility and positive outcomes of the transitional care in our study, we assume that transitional care has potential for application in SLE patients from other settings. Further studies are needed in the future.

### Limitations

Our study had several limitations. First, the research was conducted at a single center. Second, only immediate effects of intervention were measured. Therefore, there is still a need to investigate whether there is any improvement in self-care and quality of life 3 months after discharge and whether there is any decrease in readmission to hospital to determine the long-term effect of the intervention. Another limitation is that we did not collect the data on cost and this limits the ability to come to a conclusion that the intervention has decreased costs. An assumption is made that fewer readmissions reduce costs. However, further study with cost data could determine whether transitional care was cost-effective by comparing the cost of health-care utilization with the support costs of the health-care providers.

## Conclusions

This study shows that transitional care is an effective way to improve self-care and quality of life in adult patients with SLE and to reduce readmissions. However, further studies are needed to identify these effects.

## Additional file

Additional file 1: **Table S1**. Omaha System assessment-intervention framework for adult SLE patients. (DOCX 34 kb)

## Abbreviations

ANCOVA: Analysis of covariance
ESCA: Exercise of Self-Care Agency Scale
SF-36 MCS: Mental component summary of Medical Outcomes Study Short Form 36-item Health Survey
SF-36 PCS: Physical component summary of Medical Outcomes Study Short Form 36-item Health Survey
SF-36: Medical Outcomes Study Short Form 36-item Health Survey
SLE: Systemic lupus erythematosus
SLEDAI-2K: Systemic Lupus Erythematosus Disease Activity Index 2000
