# Supplementary material for: Effects of transitional care on self-care, readmission rates, and quality of life in adult patients with systemic lupus erythematosus: a randomized controlled trial
Source: Arthritis Res Ther. 2018 Aug 16;20:184. doi: 10.1186/s13075-018-1670-4 (PMC6097293; doi:10.1186/s13075-018-1670-4)
Supplement: Supplementary file 1 — Table S1. Omaha System assessment-intervention framework for adult SLE patients. (DOCX 34 kb) [file 13075_2018_1670_MOESM1_ESM.docx]

Table S1 Omaha System assessment-intervention framework for adult SLE patients

| Domain | Problem | Problem Rating Scale | Category | Interventions |
| --- | --- | --- | --- | --- |
| Physiological Domain | **1.Skin**  🗆 integrity  🗆 rash:  location , range  🗆 itch  🗆 damage:  location , range  🗆 other | Knowledge：🗆1 🗆2 🗆3 🗆4 🗆5  Behavior: 🗆1 🗆2 🗆3 🗆4 🗆5  Status: 🗆1 🗆2 🗆3 🗆4 🗆5 | 🗆 Ⅰ  🗆 Ⅱ  🗆 Ⅲ  🗆 Ⅳ | 🗆 dietary management  🗆 personal hygiene  🗆 skin care  🗆 others |
|  | **2.Pain**  🗆 no  🗆 yes:  position:  intensity:  (VAS score) | Knowledge：🗆1 🗆2 🗆3 🗆4 🗆5  Behavior: 🗆1 🗆2 🗆3 🗆4 🗆5  Status: 🗆1 🗆2 🗆3 🗆4 🗆5 | 🗆 Ⅰ  🗆 Ⅱ  🗆 Ⅲ  🗆 Ⅳ | 🗆 screening procedures  🗆 signs/symptoms-physical care  🗆 relaxation/breathing techniques  🗆 medication administration  🗆 others |
|  | **3.Oral health**  🗆 normal  🗆 mucosal ulcer  🗆 dental defects  🗆 dry mouth  🗆 mouth-bitterness  🗆 gingival bleeding/pain 🗆 other | Knowledge：🗆1 🗆2 🗆3 🗆4 🗆5  Behavior: 🗆1 🗆2 🗆3 🗆4 🗆5  Status: 🗆1 🗆2 🗆3 🗆4 🗆5 | 🗆 Ⅰ  🗆 Ⅱ  🗆 Ⅲ  🗆 Ⅳ | 🗆 anatomy/physiology  🗆 infection precautions  🗆 personal hygiene  🗆 medical/dental care  🗆 dietary management  🗆 others |
|  | **4. Infectious condition**  🗆 normal  🗆 fever  🗆 positive screening/ culture results  🗆 multiple drug -resistant bacteria infection  🗆 other | Knowledge：🗆1 🗆2 🗆3 🗆4 🗆5  Behavior: 🗆1 🗆2 🗆3 🗆4 🗆5  Status: 🗆1 🗆2 🗆3 🗆4 🗆5 | 🗆 Ⅰ  🗆 Ⅱ  🗆 Ⅲ  🗆 Ⅳ | 🗆 infection precautions  🗆 medication action/  side effects  🗆 medication administration  🗆 specimen collection  🗆 other |
| Domain | Problem | Problem Rating Scale | Category | Interventions |
| Physiological Domain | **5.Neuro-musculo-skelet-al function**  🗆 normal  🗆 myodynamia weakened  🗆 range of motion limited  🗆 walking gait disorder  🗆 other | Knowledge：🗆1 🗆2 🗆3 🗆4 🗆5  Behavior: 🗆1 🗆2 🗆3 🗆4 🗆5  Status: 🗆1 🗆2 🗆3 🗆4 🗆5 | 🗆 Ⅰ  🗆 Ⅱ  🗆 Ⅲ  🗆 Ⅳ | 🗆 anatomy/physiology  🗆 exercises  🗆 gait training  🗆 mobility/transfers  🗆 nursing care  🗆 others |
|  | **6.Respiration**  R: times per minute  🗆 normal  🗆 abnormal breathing patterns  🗆 abnormal breathing sound  🗆 cough/expectoration  🗆 polypnea  🗆 other | Knowledge：🗆1 🗆2 🗆3 🗆4 🗆5  Behavior: 🗆1 🗆2 🗆3 🗆4 🗆5  Status: 🗆1 🗆2 🗆3 🗆4 🗆5 | 🗆 Ⅰ  🗆 Ⅱ  🗆 Ⅲ  🗆 Ⅳ | 🗆 anatomy/physiology  🗆 coping skills  🗆 dietary management  🗆 exercises  🗆 infection precautions  🗆 medication administration  🗆 respiratory care  🗆 others |
|  | **7.Circulation**  P： times per minute  BP： / mmHg  🗆 normal  🗆 dysarteriotony  🗆 abnormal pulse  🗆 chest tightness/  shortness of breath  🗆 edema  🗆 others | Knowledge：🗆1 🗆2 🗆3 🗆4 🗆5  Behavior: 🗆1 🗆2 🗆3 🗆4 🗆5  Status: 🗆1 🗆2 🗆3 🗆4 🗆5 | 🗆 Ⅰ  🗆 Ⅱ  🗆 Ⅲ  🗆 Ⅳ | 🗆 anatomy/physiology  🗆 skin care  🗆 dietary management  🗆 coping skills  🗆 exercises  🗆 medication administration 🗆 others |
| Domain | Problem | Problem Rating Scale | Category | Interventions |
| Physiological Domain | **8. Urinary function**  🗆 normal  🗆 urinary tract infections  🗆 nocturia  🗆 polyuria / oliguria  🗆 proteinuria  🗆 cylindruria  🗆 other | Knowledge：🗆1 🗆2 🗆3 🗆4 🗆5  Behavior: 🗆1 🗆2 🗆3 🗆4 🗆5  Status: 🗆1 🗆2 🗆3 🗆4 🗆5 | 🗆 Ⅰ  🗆 Ⅱ  🗆 Ⅲ  🗆 Ⅳ | 🗆 anatomy/physiology  🗆 dietary management  🗆 medication administration  🗆 infection precautions  🗆 others |
|  | **9.Digestion-hydration**  🗆 normal  🗆 nausea/vomiting  🗆 anorexia/bulimia  🗆 ascites  🗆 other | Knowledge：🗆1 🗆2 🗆3 🗆4 🗆5  Behavior: 🗆1 🗆2 🗆3 🗆4 🗆5  Status: 🗆1 🗆2 🗆3 🗆4 🗆5 | 🗆 Ⅰ  🗆 Ⅱ  🗆 Ⅲ  🗆 Ⅳ | 🗆 anatomy/physiology  🗆 screening procedures  🗆 dietary management  🗆 medication action/side effects  🗆 coping skills  🗆 others |
| Psychosocial Domain | **1.** **Mental health**  🗆 normal  🗆 nervous/irritable  🗆 anxious  🗆 depressed  🗆 lose interest in activity/self-care  🗆 other | Knowledge：🗆1 🗆2 🗆3 🗆4 🗆5  Behavior: 🗆1 🗆2 🗆3 🗆4 🗆5  Status: 🗆1 🗆2 🗆3 🗆4 🗆5 | 🗆 Ⅰ  🗆 Ⅱ  🗆 Ⅲ  🗆 Ⅳ | 🗆 anatomy/physiology  🗆 anger management  🗆 stress management  🗆 coping skills  🗆 relaxation/breathing techniques  🗆 support group  🗆 nursing care  🗆 others |
|  | **2. Neglect**  🗆 no  🗆 lack of physical care  🗆 lack of emotional support  🗆 lack of medical care  🗆 other | Knowledge：🗆1 🗆2 🗆3 🗆4 🗆5  Behavior: 🗆1 🗆2 🗆3 🗆4 🗆5  Status: 🗆1 🗆2 🗆3 🗆4 🗆5 | 🗆 Ⅰ  🗆 Ⅱ  🗆 Ⅲ  🗆 Ⅳ | 🗆 support system  🗆 support group  🗆 community outreach worker services  🗆 other |

| Domain | Problem | Problem Rating Scale | Category | Interventions |
| --- | --- | --- | --- | --- |
| Psychosocial Domain | **3. Social contact**  🗆 normal  🗆 lack of sociability  🗆 few outdoor/leisure activities  🗆 other | Knowledge：🗆1 🗆2 🗆3 🗆4 🗆5  Behavior: 🗆1 🗆2 🗆3 🗆4 🗆5  Status: 🗆1 🗆2 🗆3 🗆4 🗆5 | 🗆 Ⅰ  🗆 Ⅱ  🗆 Ⅲ  🗆 Ⅳ | 🗆 communication  🗆 interaction  🗆 behavior modification  🗆 discipline  🗆 others |
|  | **4. Interpersonal relationship**  🗆 normal  🗆 lack of communication skills  🗆 interpersonal tension  🗆 other | Knowledge：🗆1 🗆2 🗆3 🗆4 🗆5  Behavior: 🗆1 🗆2 🗆3 🗆4 🗆5  Status: 🗆1 🗆2 🗆3 🗆4 🗆5 | 🗆 Ⅰ  🗆 Ⅱ  🗆 Ⅲ  🗆 Ⅳ | 🗆 communication  🗆 interaction  🗆 discipline  🗆 others |
| Health-related Behaviors Domain | **1.** **Sleep and rest patterns**  🗆 normal  🗆 difficulty to fall asleep  🗆 dreaminess  🗆 easy to wake  🗆 early to wake  🗆 difficult to fall asleep after waking up  🗆 other | Knowledge：🗆1 🗆2 🗆3 🗆4 🗆5  Behavior: 🗆1 🗆2 🗆3 🗆4 🗆5  Status: 🗆1 🗆2 🗆3 🗆4 🗆5 | 🗆 Ⅰ  🗆 Ⅱ  🗆 Ⅲ  🗆 Ⅳ | 🗆 rest/sleep  🗆 relaxation/breathing techniques  🗆 behavior modification  🗆 medication action/side effects  🗆 others |
|  | **2. Medication regimen**  🗆 normal  🗆 non-adherence to medication recommendation  🗆 occurrence of side effects  🗆 inappropriate drug storage  🗆 other | Knowledge：🗆1 🗆2 🗆3 🗆4 🗆5  Behavior: 🗆1 🗆2 🗆3 🗆4 🗆5  Status: 🗆1 🗆2 🗆3 🗆4 🗆5 | 🗆 Ⅰ  🗆 Ⅱ  🗆 Ⅲ  🗆 Ⅳ | 🗆 medication action/side effects  🗆 medication administration  🗆 medication coordination/ ordering  🗆 medication set-up  🗆 other |
| Domain | Problem | Problem Rating Scale | Category | Interventions |
| Health-related Behaviors Domain | **3. Nutrition**  Weight: kg  Height: m  🗆 normal  🗆 non-adherence to diet guidance  🗆 malnutrition  🗆 unsatisfactory blood glucose control  🗆 other | Knowledge：🗆1 🗆2 🗆3 🗆4 🗆5  Behavior: 🗆1 🗆2 🗆3 🗆4 🗆5  Status: 🗆1 🗆2 🗆3 🗆4 🗆5 | 🗆 Ⅰ  🗆 Ⅱ  🗆 Ⅲ  🗆 Ⅳ | 🗆 behavior modification  🗆 dietary management  🗆 exercises  🗆 others |
|  | **4. Personal care**  🗆 take care of oneself  🗆 unwilling / unable / forgetting to complete personal care  🗆 other | Knowledge：🗆1 🗆2 🗆3 🗆4 🗆5  Behavior: 🗆1 🗆2 🗆3 🗆4 🗆5  Status: 🗆1 🗆2 🗆3 🗆4 🗆5 | 🗆 Ⅰ  🗆 Ⅱ  🗆 Ⅲ  🗆 Ⅳ | 🗆 caretaking skills  🗆 support group  🗆 nursing care  🗆 others |
|  | **5.** **Substance use**  🗆 no  🗆 abusing over-the-  counter drugs  🗆 smoking  🗆 drinking  🗆 other | Knowledge：🗆1 🗆2 🗆3 🗆4 🗆5  Behavior: 🗆1 🗆2 🗆3 🗆4 🗆5  Status: 🗆1 🗆2 🗆3 🗆4 🗆5 | 🗆 Ⅰ  🗆 Ⅱ  🗆 Ⅲ  🗆 Ⅳ | 🗆 substance use cessation  🗆 education  🗆 behavior modification  🗆 coping skills  🗆 others |
|  | **6. Physical activity**  🗆 normal  🗆 sedentary lifestyle  🗆 lack of exercise  🗆 inappropriate form/ intensity of exercise  🗆 other | Knowledge：🗆1 🗆2 🗆3 🗆4 🗆5  Behavior: 🗆1 🗆2 🗆3 🗆4 🗆5  Status: 🗆1 🗆2 🗆3 🗆4 🗆5 | 🗆 Ⅰ  🗆 Ⅱ  🗆 Ⅲ  🗆 Ⅳ | 🗆 screening procedures  🗆 education  🗆 behavior modification  🗆 exercises  🗆 mobility/transfers  🗆 other |

| Domain | Problem | Problem Rating Scale | Category | Interventions |
| --- | --- | --- | --- | --- |
| Health-related Behaviors Domain | **7. Family planning**  🗆 normal  🗆 do not understand the relationship between disease and pregnancy  🗆 do not understand the timing of pregnancy  🗆 not contraception / inappropriate contraception  🗆 other | Knowledge：🗆1 🗆2 🗆3 🗆4 🗆5  Behavior: 🗆1 🗆2 🗆3 🗆4 🗆5  Status: 🗆1 🗆2 🗆3 🗆4 🗆5 | 🗆 Ⅰ  🗆 Ⅱ  🗆 Ⅲ  🗆 Ⅳ | 🗆 family planning care  🗆 behavior modification  🗆 discipline  🗆 others |
|  | **8. Health care**  **supervision**  🗆 failure to seek assessment / treatment as the symptoms require  🗆 failure to adhere to revisit advice of healthcare providers  🗆 other | Knowledge：🗆1 🗆2 🗆3 🗆4 🗆5  Behavior: 🗆1 🗆2 🗆3 🗆4 🗆5  Status: 🗆1 🗆2 🗆3 🗆4 🗆5 | 🗆 Ⅰ  🗆 Ⅱ  🗆 Ⅲ  🗆 Ⅳ | 🗆 continuity of care  🗆 wellness  🗆 communication  🗆 signs/symptoms-physical  🗆 others |
| Environmental Domain | **1.** **Sanitation**  🗆 clean  🗆 improper storage of food  🗆 residence dirty mess  🗆 poor ventilation  🗆 other | Knowledge：🗆1 🗆2 🗆3 🗆4 🗆5  Behavior: 🗆1 🗆2 🗆3 🗆4 🗆5  Status: 🗆1 🗆2 🗆3 🗆4 🗆5 | 🗆 Ⅰ  🗆 Ⅱ  🗆 Ⅲ  🗆 Ⅳ | 🗆 homemaking/  housekeeping  🗆 home  🗆 environment  🗆 others |
|  | **2. Neighborhood/**  **workplace safety**  🗆 normal  🗆 insufficient space/  resources to promote  health  🗆 physical hazards  🗆 chemical hazards  🗆 other | Knowledge：🗆1 🗆2 🗆3 🗆4 🗆5  Behavior: 🗆1 🗆2 🗆3 🗆4 🗆5  Status: 🗆1 🗆2 🗆3 🗆4 🗆5 | 🗆 Ⅰ  🗆 Ⅱ  🗆 Ⅲ  🗆 Ⅳ | 🗆 other community resources  🗆 environment  🗆 safety  🗆 other |

Knowledge：1=No knowledge; 2=Minimal knowledge; 3=Basic knowledge;

4=Adequate knowledge; 5=Superior knowledge

Behavior：1=Not appropriate behavior; 2=Rarely appropriate behavior; 3=Inconsistently appropriate behavior; 4=Usually appropriate behavior; 5=Consistently appropriate behavior

Status：1=Extreme signs/ symptoms; 2=Severe signs/ symptoms; 3=Moderate signs/ symptoms; 4=Minimal signs/ symptoms; 5=No signs/ symptoms

Ⅰ=*Teaching, Guidance, and Counseling*: Series of activities designed to provide information and materials, encourage action and responsibility for self-care and coping.

Ⅱ= *Treatments and Procedures*: Series of technical activities designed to prevent, decrease, or alleviate signs and symptoms of the individual.

Ⅲ= *Case Management*: Series of activities designed to improve communication between different health care providers, as well as between health care providers and patients.

Ⅳ= *Surveillance*: Series of activities designed to identify the individual's status in relation to a given condition.
